# Academic performance of K-12 students in an online-learning environment for mathematics increased during the shutdown of schools in wake of the COVID-19 pandemic

**DOI:** 10.1371/journal.pone.0255629

**Published:** 2021-08-03

**Authors:** Markus Wolfgang Hermann Spitzer, Sebastian Musslick

**Affiliations:** 1 Albert-Ludwigs-Universität Freiburg, Freiburg, Germany; 2 Princeton Neuroscience Institute, Princeton University, Princeton, NJ, United States of America; ISI Foundation, ITALY

## Abstract

The shutdown of schools in response to the rapid spread of COVID-19 poses risks to the education of young children, including a widening education gap. In the present article, we investigate how school closures in 2020 influenced the performance of German students in a curriculum-based online learning software for mathematics. We analyzed data from more than 2,500 K-12 students who computed over 124,000 mathematical problem sets before and during the shutdown, and found that students’ performance increased during the shutdown of schools in 2020 relative to the year before. Our analyses also revealed that low-achieving students showed greater improvements in performance than high-achieving students, suggesting a narrowing gap in performance between low- and high-achieving students. We conclude that online learning environments may be effective in preventing educational losses associated with current and future shutdowns of schools.

## 1. Introduction

The COVID-19 pandemic led to a sudden shutdown of schools in 2020, affecting more than 1.6 billion students in over 190 countries (UNESCO 2020 [32]). The shutdown required teachers, students and parents to rapidly adopt to a new homeschooling situation, lasting from a few weeks to several months, depending on governmental policy. While the full consequences of this outage will take years or even decades to unfold, preliminary data highlight immediate effects on student’s academic performance and well-being: the COVID-19 pandemic required students to be educated from home which has been linked to lower performance on national tests [1–10] (note that reference [10] is pending peer-review), higher stress and anxiety [2, 3], lower sleep quality [4], as well as a general decrease in student’s wellbeing [5–7]. However, little is known about the pandemic’s impact on the performance of K-12 students in online learning environments—educational tools that became popular complements to traditional classroom work over the past years. Here, we seek to investigate whether the performance of K-12 students in mathematics—quantified in terms of error rate and difficulty of assigned problem sets in an online learning environment—changed during the shutdown of schools in wake of the COVID-19 pandemic.

Preliminary studies investigating the effects of the shutdown paint a negative picture, suggesting a detrimental influence on academic performance and general wellbeing. For instance, scores on national exams in the Netherlands have been found to decrease by three percentile points after the shutdown of schools compared to the years before [8]. Another study, involving students in Germany, reported that general screen time (time spent on television, computer games or social media) increased by more than one hour a day and study time was cut in half as a consequence of the school closures [9]. Reduced study time has been linked to significant decreases in curriculum-based learning for children, adolescents and young adults, as families report to struggle with educating their children at home [10]. Finally, more time spent at home has been linked to increased rates of child abuse [6, 7].

An important consequence of school shutdowns concerns an increase in educational differences between students in the same cohort [8, 11]. A recent study from the UK found that children from low-income families are less likely to participate in online classes, are spending 30% less time learning at home, and have limited access to educational resources [10]. These inequalities in learning styles are reported to widen achievement disparities between low-performing and high-performing students [8, 10, 11]. Another study, based on 55 million library check-outs in Denmark, showed that families with higher socioeconomic status (SES) borrowed more books than families with lower SES, and that this difference increased during the shutdown of schools [12]. Given positive correlations between SES and mathematical achievements, as well as higher salaries during adulthood [13] such differential effects on the education of students with different SES are troubling—especially in light of the “Every Student Succeeds Act” [14] which aims to provide equal opportunities for students in poverty, for minorities, students with limited language skills, and those who need special education in the United States. Results from these studies comport with the observation that students with a small advantage in academic performance continue to benefit, while those with a slight disadvantage continue to lose ground, as has been reported for reading [15], vocabulary acquisition [16] and mathematics [17–19]. This observation, commonly referred to as the *Matthew effect*—referencing the biblical assertion “the rich get richer” in the Gospel of Matthew [20, 21]—can be attributed to an interaction between motivational beliefs and the capacity for self-regulated learning [22, 23]. For instance, theories of self-regulated learning suggest that students with lower skills, e.g., in mathematics, may adopt maladaptive beliefs about their own self-efficacy in learning, hampering further achievement [24, 25]. Mitigating the Matthew effect in the midst of homeschooling situation precipitated by the pandemic may require adaptive educational practices that minimize performance differences between low-performing and high-performing students [17].

Online learning platforms provide solutions to the new homeschooling situation and concomitant demands for remote teaching. Here, we analyzed data of a curriculum-based (grades four to ten) online learning software for mathematics, used within the class context as a complement to traditional classroom work (see methods for a detailed description of the software and data collection). Its use increased remarkably during the school closures, with three times more students who studied with the software (see S1 Fig in S1 File), reflecting the need for online teaching methods in lieu of traditional teaching at school. In this study, we analyzed data from this learning software, to investigate the effects of school closures on the performance of students in problem sets assigned by their teachers before and during the shutdown of schools in Germany. We also examined potential effects on changes in performance depending on how well students performed before the shutdown.

Based on a growing number of studies reporting detrimental effects of the pandemic on students’ performance and well-being (see above), we hypothesized that the academic performance of K-12 students decreased during the school closures in 2020 relative to the previous year. To test this hypothesis, we analyzed differences in the absolute error rate of students on mathematical problem sets between 2020 and 2019. We analyzed these differences in a within-group analysis (Analysis 1a) controlling for the number of problem sets each student computed, the number of repetitions on each problem set, and overall experience with the software. Since absolute error rate can vary as a function of problem set difficulty, we also assessed how school closures affected the error rate of students *relative* to a reference group (relative error rate), using the same within-group cohort (Analysis 1b). We also conducted a within-group analysis to examine whether problem sets assigned by teachers were associated with a lower difficulty during the shutdown compared to the previous year. Finally, according to the Matthew effect, high-performing should be less affected by this than low-performing students, resulting in a widening performance gap. Thus, we expected that students with comparably low performance in 2019 would show greater performance decrements as a consequence of school closures in 2020, relative to students with comparably high performance in 2019, suggesting a widening performance gap between students. To test this hypothesis, we assessed the average relative error rate of each student in 2020 as a function their average relative error rate in 2019, controlling for number of problem set assignments and problem set repetitions (Analysis 2). To foreshadow results from these analyses, we observed—contrary to our expectations—a decrease in students’ error rate *and* relative error rate, reflecting higher performance during the shutdown of schools in 2020 compared to the same time frame in 2019. In addition, we observed a decrease in performance differences between low-performing students and high-performing students from 2019 to 2020.

## 2. Methods

### 2.1. Software

**Fig 1:**
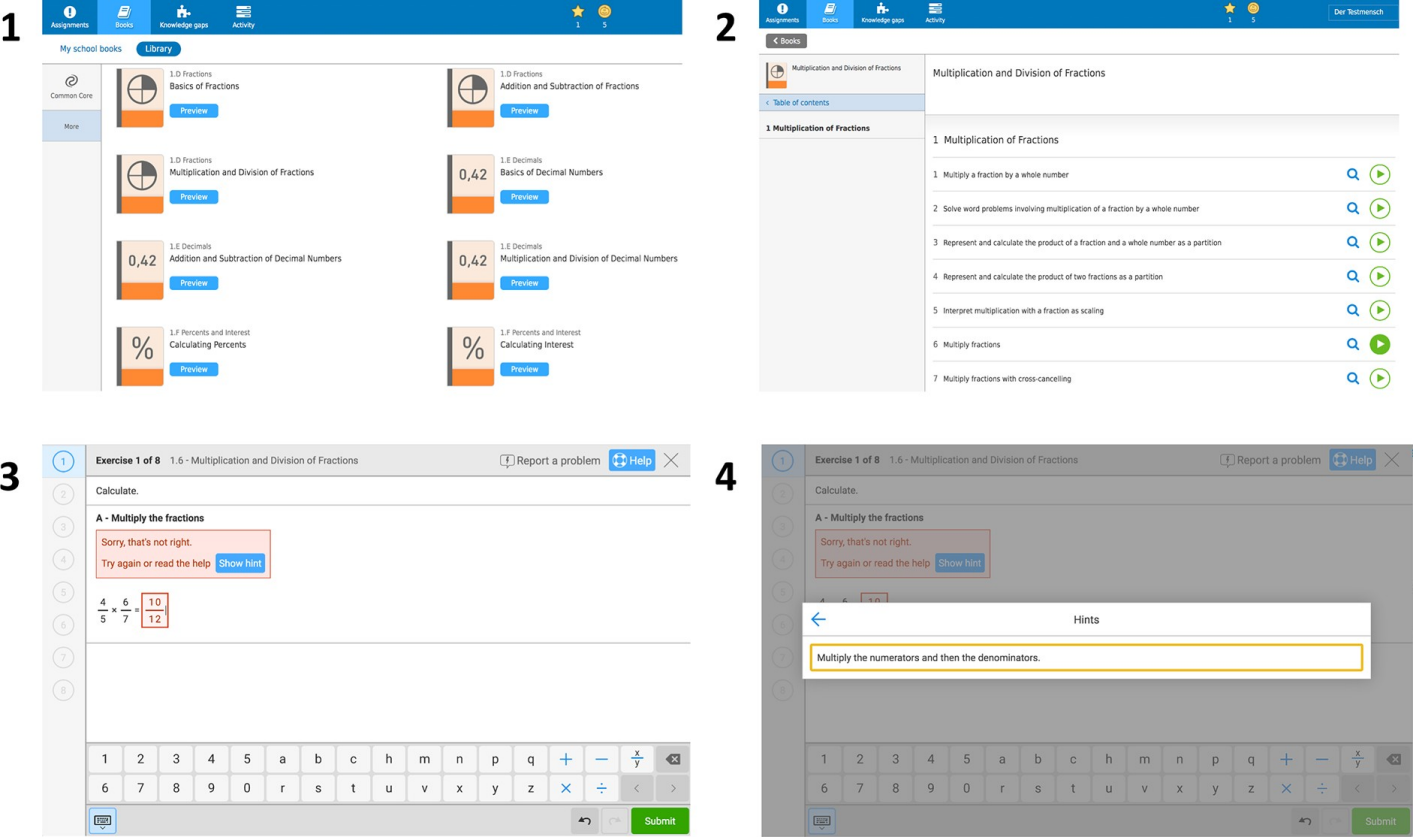
Interface for assignments und their execution in Bettermarks. (1) Teachers and students can select from a library with over 100 different books. Each book contains an introduction as well as different mathematical problem sets. (2) A problem set contains several individual problems. (3) When students compute a problem and submit an answer, they receive immediate feedback on whether their answer is right or wrong. Students have two attempts on each problem. (4) Students may request up to one hint when computing a problem.

The Bettermarks software has been distributed to schools in 2008 and covers the curricula of mathematics in Germany from classes 4–10, with 100 book topics (i.e., more general themes, such as “Basic calculations of percentages”, or “Advanced calculations of fractions”). The software comprises book topics from a variety of mathematical topics such as number theory, algebra, combinations, geometry, probability, and statistics. In addition, problem sets cover various mathematical competencies including (but not limited to) solving equations, simplifying equations, retrieval of mathematical laws and plotting (see S2 Fig in S1 File). The software is distributed over all states in Germany and used in different types of schools, such as public schools (Gymnasium, Realschule, Hauptschule, and Gesamtschule) and private schools. Thus, the students who use Bettermarks may represent the average population of students in Germany. A book topic provides the student with an introduction to the topic and includes between four and 21 problem sets, each of which entails eight individual problems on average. The software can be used as a complement to a traditional curriculum in mathematics. The bettermarks software is used to practice mathematical problem sets. Teachers use the software to assign problem sets which students may compute in class (in the event that teachers provide them with time to compute these problem sets during their classes), or at home as homework assignments. Teachers may also use the software as an additional practice tool on top of other pencil paper assignments. Fig 1 illustrates the interface of Bettermarks, including the selection of book topics, the selection and calculation of problem sets, as well as mechanisms for feedback. Please see S2 Fig in S1 File for more problem set examples on other mathematical topics. Problem sets can be assigned in two ways: (a) teachers assign problem sets to students, or (b) students self-select their own problem sets. In this study, we restricted our analysis to problem sets assigned by schoolteachers. Independent of the assignment policy, students receive feedback regarding their accuracy on computed problem sets and may request up to one hint for solving a problem. Students may repeat a problem set; however, the parameterization of individual problems changes with every new attempt. If students receive negative feedback on their first attempt of a problem within a problem set, they can make a second attempt on that problem. The collected data includes information about (a) which problem set was computed, (b) the number of distinct problem sets that each student computed for a given book topic, and (c) the number of times a student repeated a given problem set. Teachers registered themselves and their students with pseudonyms at the learning platform and thus, only anonymized data was collected from students (for another detailed description of the software see [26]).

### 2.2. Identification strategy

We restricted analyses to two distinct groups of students. For the within-group analysis, we considered a group of German students who used the software both before the shutdown (March 15^th^, 2019 until June 15^th^, 2019) and during the shutdown (March 15^th^, 2020 until June 15^th^, 2020). This group comprised data of 2,556 students (314 school classes) who calculated a total of 124,597 problem sets (1,234 unique problem sets). All problem sets that these students computed were assigned by their teachers as homework. Due to the anonymization requirements of the learning software, we did not include any demographic information into our analysis. Thus, the sample was composed of students from all states in Germany and all types of schools from grades 4 to10.

The second group of students was included as a reference group for problem set difficulty. This group comprised data from 10,693 students (1,373 classes) who used the software before the shutdown (March 15^th^, 2019 until June 15^th^, 2019) but stopped using the software afterwards. The reference group calculated a total of 209,294 problem sets (1,093 unique problem sets). All problem sets that these students computed were assigned by their teachers as homework. S3 Fig in S1 File illustrates the overall software usage by the students across the two time windows, depicting the distribution of problem sets computed per day.

For each of these groups and each time window, we only included students who computed more than 10 problem sets. In addition, we ensured that each problem set was computed by at least 20 students per group and time window.

### 2.3. Independent and dependent variables

We considered four independent variables for the analyses reported below. These comprised a categorial variable encoding the time window (labeled *time window*; time window 1: March 15^th^, 2019 –June 15^th^, 2019; time window 2: March 15^th^, 2020—June 15^th^, 2020), to compare the effect of the shutdown with a similar time period in the previous year. In addition to time window, we considered three covariates: the number of times a student repeated a given problem set (labeled *repetitions;* treated as interval variable), the total number of assignments a student computed (labeled as *assignments;* treated as interval variable), as well as the cumulative number of assignments a student computed so far (labeled as *cumulative assignments*; treated as interval variable).

We assessed three performance-related dependent variables to investigate the impact of the school closures. First, we computed student’s *absolute error rate* on each problem set. However, problem sets can vary in terms of their difficulty, and the absolute error rate can depend on the difficulty of a problem set. Thus, we also sought to compute students’ error rate relative to the difficulty of a problem set, and refer to this metric as *relative error rate* (see below). Since the difficulty of a problem set cannot be easily determined in an objective manner, we computed a performance-based proxy for *problem set difficulty*. We operationalized the difficulty for a given problem set as the average error rate with which a reference group computed that problem set. We then determined the relative error rate of a student on a given problem set as the difference between their absolute error rate on that problem set and the average error rate of the reference group on that problem set. A negative relative error rate indicates that a student performed better on the problem set relative to the reference group. Conversely, a positive relative error rate suggests that the student performed worse compared to the reference group. Please note that the average error rate of each problem set of the reference group was determined based on the time frame before the pandemic, from March 15^th^, 2019 to June 15^th^, 2019. Finally, to investigate whether teachers assigned more or less difficult problem sets during the pandemic relative to the year before, we assessed problem set difficulty as a function of time. As noted above, the average error rate on each problem set of the corresponding reference group, determined between March 15^th^, 2019 and June 15^th^, 2019, served as a proxy for problem set difficulty. That is, if teachers assigned problem sets in which the reference group yielded high error rates in 2019, this would indicate that assigned problem sets were rather difficult. Conversely, if teachers assigned problem sets in which the reference group yielded low error rates in 2019, this would indicate that assigned problem sets were less difficult.

### 2.4. Data analysis

The statistical analysis was conducted in the R environment for statistical computing. Each analysis involved fitting a linear mixed model to the data, using the lmerTest package [27]. For each within-group analysis, fixed effects comprised the categorical factor time window (2019/2020), and the two continuous factors repetitions, and assignments. We treated classes and students as random effects by including a nested random intercept for classes and students. In addition, we included a random slope for classes and students with respect to time window, to account for individual differences in the effect of time window on error rate. As noted above, we considered three different dependent variables for the within-group analyses: absolute error rate (Analysis 1a), relative error rate (Analysis 1b) and assigned problem set difficulty (Analysis 1c), resulting in three different statistical models.

We expected students’ absolute and relative error rates to increase during the second time window in which schools were shut down, as indicated by positive regression slopes for the factor time window. In addition, we expected that more repetitions on a problem set, as well as more computed assignments would yield lower absolute and relative error rates for a given student, as would be reflected by negative regression slopes for the repetitions and assignments variables in Analyses 1a-b. In addition, we expected students to perform better with more software usage due to habituation effects, indicated by a negative regression slope for cumulative assignments. Finally, we expected teachers to assign problem sets with a low difficulty to students during the shutdown of schools as compared to the year before, as would be reflected by a negative regression slope for time window in Analyses 1c. We had no expectations for the influence of the repetitions and assignments variables on assigned problem set difficulty.

In a final analysis (Analysis 2), we examined whether the shutdown differentially affected changes in the performance of low-performing and high-performing students with a linear regression. Since the performance measurement was continuous, we define low-performing students as students with a relative error rate above zero (worse than the average of the reference group) and high-performing students with a relative error rate below zero. To investigate changes in the performance difference between low-performing and high-performing students, we regressed the average relative error rate of each student in 2019 against their average relative error rate in 2020, using the same time windows as reported above. To control for differences in the number of problem set assignments and problem set repetitions across students and time windows, we computed the average difference in number of assignments between the two time windows (*assignment difference*) and the average difference in number of repetitions (*repetition difference*) between the two time windows for each student, and included both variables in the regression model.

**Fig 2:**
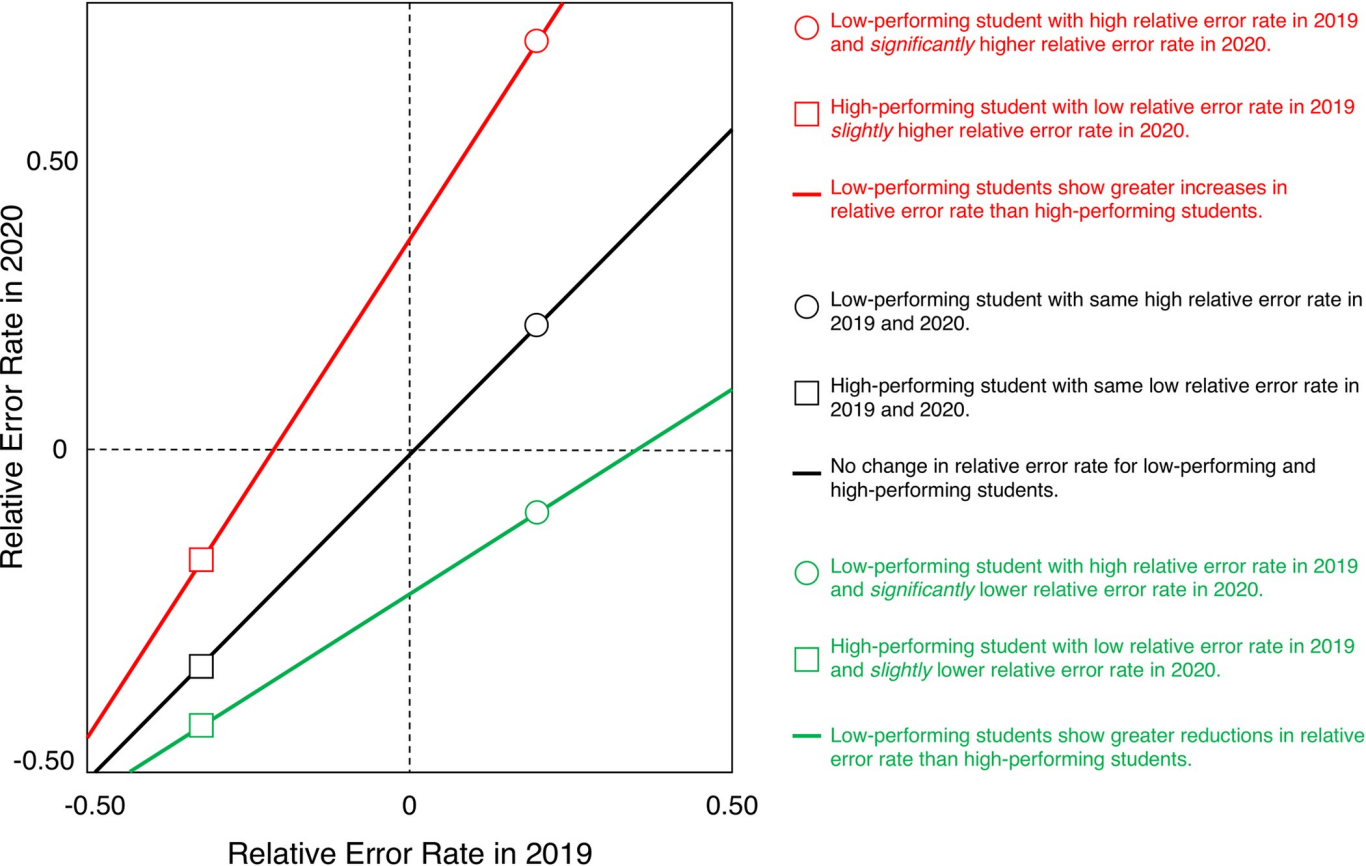
Hypothetical regression analysis of relative performance. Students’ relative error rate in 2020 (ordinate) is regressed against their relative error rate in 2019 (abscissa). Three different hypothetical outcomes are illustrated in red, black and green (see legend and text).

Fig 2 depicts three different hypothetical outcomes of this analysis. As noted above, the sign of a student’s relative error rate in 2019 indicates whether they performed better (negative relative error rate) or worse (positive relative error rate) on problem sets, relative to a reference group. Thus, students who performed better than the reference group in 2019 are located at the left side of the abscissa in Fig 2, whereas students who performed worse than the reference group in 2019 are located on the right side of the abscissa. Analogously, low-performing students in 2020 are located on the upper part of the ordinate, whereas high-performing students are located on the lower part of the ordinate. The black line in Fig 2 denotes the null-hypothesis (identity function), proposing that the relative error rate of all students did not change between 2019 and 2020. The green line illustrates a narrowing of performance differences between students: low-performing students show greater reductions in relative error rate than high-performing students, resulting in a negative regression intercept and a regression slope of less than 1. Conversely, the red line exemplifies the Matthew effect: high-performing students in 2019 show greater reductions in relative error rate than low-performing students resulting in a positive regression intercept and a regression slope of greater than 1. We expected that (a) student’s overall relative error rate would increase from 2019 to 2020, as indicated by a positive regression intercept, and that (b) low-performing students showed greater increases in relative error rates than high-performing students, as indicated by a regression slope greater than 1 (see red line in Fig 2).

Note that the performed analysis may be subject to regression dilution bias, that is, a biasing of the regression slope towards zero [28]. Such a bias can occur in the presence of measurement noise associated with both the predictor and the dependent variable [29]. Following the suggestion of a reviewer, we computed the 95% confidence interval for the corrected regression coefficient after performing a simple regression of students’ relative error rate in 2019 against their relative error rate in 2020, without consideration of co-variates [30] (assuming M = ∞). While this correction—like other methods [28, 31]—does not require explicit knowledge of the measurement noise, it relies on the simplifying assumption that the measurement noise of the regressor and regressand are uncorrelated. However, since the relative error rate of the same students in 2019 and 2020 represent the same type of measurement performed at two different time points, it is possible that the measurement noise in both variables is correlated, potentially violating the assumption of [30]. Thus, the estimated correction must be interpreted under consideration of a potential violation of this assumption.

## 3. Results

**Fig 3:**
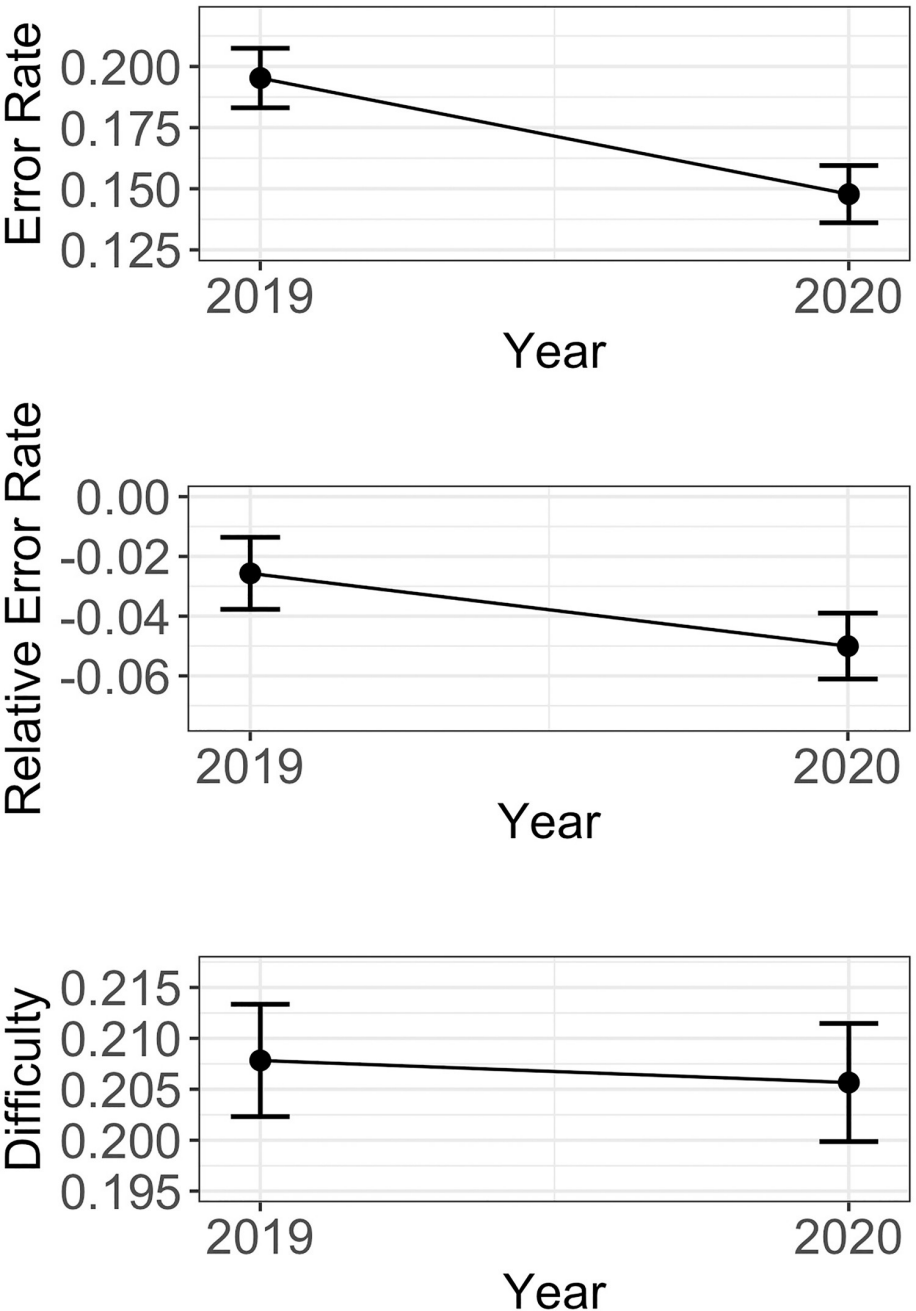
Estimates of absolute error rate, relative error rate and problem set difficulty as a function of time. Results from the Analyses 1a-c are depicted. Error rates and relative error rates significantly decreases during the shutdown compared to the same time in the previous year. There was no significant difference in problem set difficulty between the two time windows. Points indicate mean estimates, error bars indicate the standard errors of the mean across students. Connected lines denote that both time windows include results from the same students.

**Fig 4:**
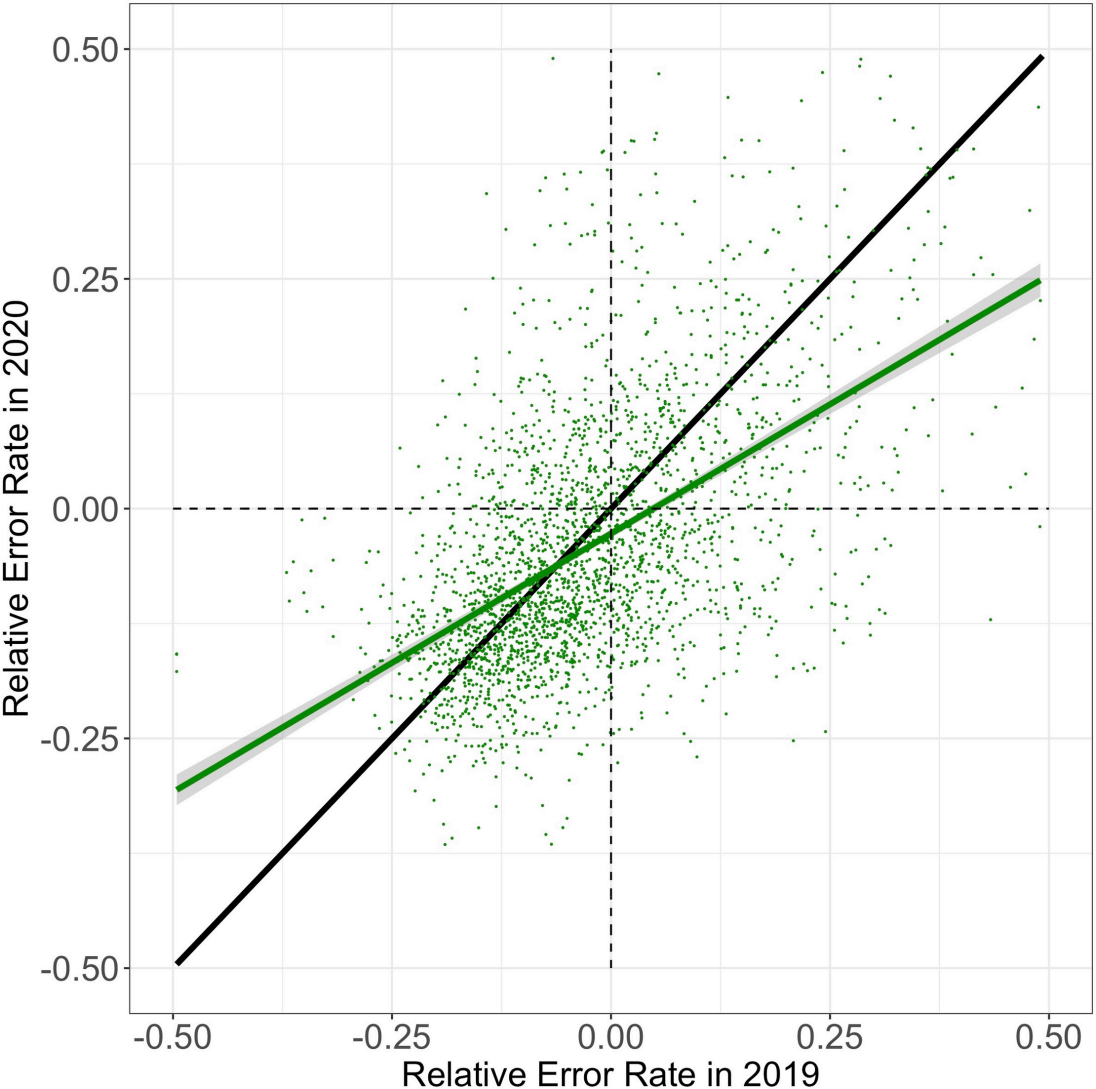
Relative error rate in 2020 as a function of relative error rate in 2019. Each data point corresponds to a student, showing their average relative error rate in 2019 (abscissa) and 2020 (ordinate). The green line corresponds to a linear regression fitted to the data. Grey shades indicate the standard error of the mean across students. The black line depicts the identify function (intercept of 0 with a slope of 1) for reference. The intercept of the regression (green) is below zero, indicating that the relative error rate of students decreased from 2019 to 2020. The slope of the regression is below 1, indicating that students categorized as low-performing in 2019 showed greater decrements in relative error rate than students categorized as high-performing (cf. Fig 2), suggesting a narrowing performance gap between students.

Results from Analyses 1 are summarized in Fig 3 (see S4 Fig in S1 File for monthly descriptive statistics of each dependent variable from January 1^st^, 2019 until June 15^th^, 2020) and results from Analysis 2 are depicted in Fig 4. Below, we describe individual effects observed in each of these analyses.

### Analysis 1a: Absolute error rate as a function of time window, repetitions, assignments, and cumulative assignments

The absolute error rate of students did significantly differ between the two time windows (*b* = -2.37e-02; *t* = -8.39; *p* < .001), with lower absolute error rates during the shutdown than before the shutdown. More repetitions and more total assignments led to lower absolute error rates (repetitions: *b* = -2.49e-02; *t* = -32.59; *p* < .001; assignments: *b* = -1.83e-03; *t* = -12.63; *p* < .001). Against our expectations, more cumulative assignments led to higher absolute error rates (*b* = 8.58e-04; *t* = 20.45; *p* < .001).

### Analysis 1b: Relative error rate as a function of time window, repetitions, assignments and cumulative assignments

Contrary to our expectation, the relative error rate significantly decreased by 2.43% during the school shutdown compared to the same time window in the previous year (*b* = -1.21e-02; *t* = -5.06; *p* < .001). Students who repeated more problem sets yielded a lower relative error rate than students who engaged in less repetitions (*b* = -4.21e-2; *t* = -61.47; *p* < .001). Students who computed more assignments yielded a lower relative error rate (*b* = -1.56e-3; t = -11.24; *p* < .001). Unexpectedly, more cumulative assignments yielded a higher relative error rate indicating no habituation effect (*b* = 1.03e-4; t = 2.70; *p* = .006).

### Analysis 1c: Problem set difficulty as a function of time window, repetitions, assignments and cumulative assignments

There was no statistically significant difference between the two time windows on problem set difficulty (*b* = -1.08e-03; *t* = -0.56; *p* = .574). Problem sets that were repeated more frequently were associated with a higher problem set difficulty (*b* = 1.46e-02; *t* = 38.19; *p* < .001). There was no significant effect of the number of computed problem sets on assigned problem set difficulty (*b* = -4.09e-05; *t* = -1.50; *p* = .134). A higher number of completed assignments was associated with higher problem set difficulties (*b* = 4.05e-04; *t* = 24.06; *p* < .001).

### Analysis 2: Changes in performance gap between students

Consistent with Analyses 1 and 2, the regression model yielded a negative intercept for relative error rate (*b = -*2.29e-02; *t* = -8.49; *p* < .001; see Fig 4), indicating that the relative error rate of students decreased in 2020 relative to the year before. In addition, and also contrary to our expectation, the regression coefficient for time window is below 1 (*b* = .56; *t* = 34.02; *p* < .001), suggesting that low-performing students in 2019 showed greater decrements in relative error rate than high-performing students. Finally, a higher number of problem set assignments and repetitions in 2020 compared to 2019 lead to a lower relative error rates in 2020 (assignments: *b* = -5.67e-02; *t* = -12.11; *p* < .001; repetitions: *b* = -4.01-e03; *t* = -3.31; *p* < .001).

A simple regression without covariates revealed a significant regression intercept with a negative coefficient of *b* = -0.03 (t = -10.34; p < .001), indicating that students with an average error rate in 2019 had a lower relative error rate in 2020. The uncorrected regression slope is *b* = 0.55 (t = -32.19; p < .001), and the 95% confidence interval for the corrected regression coefficient is estimated to lie between (0.30, 0.99). Thus, the corrected regression coefficient (below 1.0) indicates a narrowing performance gap.

## 4. Discussion

In this study, we examined the impact of the school closures on the performance of K12 students in an online learning environment for mathematics—building on a large dataset compromising over 2,500 K-12 students and over 124,000 computed problem sets—by contrasting students’ performance before the shutdown against their performance during the shutdown. The within-group analyses conducted in this study suggest that students’ performance in mathematics *improved* during the shutdown of schools relative to the year before. The suggested improvements are further evidenced by the observation that teachers assigned more difficult problem sets to students during the school closures as compared to the same time frame in 2019. Finally, the data indicate a narrowing performance gap between students: performance improvements were higher for students categorized as low-performing in 2019 compared to students categorized as high-performing. Altogether, the analyses reported in this study suggests that the shutdown of schools in wake of the COVID-19 pandemic had no detrimental effect on the performance of students in an online learning environment for mathematics.

Results from this study stand in contrast to earlier findings showing mostly detrimental effects of school closures on student’s performance and wellbeing [2–6, 8, 32]. Yet, the present study is not the first to demonstrate that students’ performance can improve during the shutdown of schools in 2020. For instance, Gonzalez and colleagues (2020) [33] analyzed the performance of students on weekly examinations in an online learning class on metabolism and found that students performed better during the shutdown of their University, relative to two cohorts of students who took the same online class in the preceding two years. In addition, they found that more students passed the course, and more students completed their assignments during the shutdown compared to the previous two years. The authors attribute this increase in performance to higher consistency in studying during the shutdown compared to the preceding years. However, the study of Gonzalez and colleagues differs from the present study with respect to students’ age and educational context (college students vs. K-12 students) and subject (metabolism vs. mathematics).

Performance improvements of students in online-learning environments, as observed in this study, could be caused by several factors. First, the performance of students may have improved within the software due to increased usage of similar educational online platforms during the pandemic [34–37]. This is evidenced by two independent meta-analyses reporting that more exposure to online-learning environments can lead to increases in the academic achievement of high school students [38, 39]. A similar effect has been observed for college students [40]. Yet, we observed performance improvements despite accounting for the amount of software usage in our regression analyses. Second, performance improvements may be driven by higher incentives provided by the teachers during the pandemic relative to the year before. Motivational theories of effort allocation suggest a link between incentivization and academic performance [41–43]. Thus, it is possible that the observed improvements in performance stem from higher incentives provided by the teachers during the pandemic relative to the year before. If this was the case, then higher incentives might have affected low-performing students to a larger extend than high-performing students, as indicated by a narrowing performance gap between students. However, prior studies suggests the opposite, demonstrating that incentives have greater effects on high-performing students compared to low-performing students [44, 45]. Thus, it remains controversial whether the increased performance of low-performing students in this study can be attributed to higher incentives. Third, it may be that students who used online learning software at home received more tutoring from their parents or caregivers, clouding the authenticity of returned homework assignments. While increased help from parents and caregivers may explain the overall positive effect of school closures observed in this study, further examination is needed to explain why low-performing students showed greater improvements in performance during homeschooling compared to high-performing students. Another potential factor explaining improvements in mathematics during school closures may be rooted in math anxiety, i.e., the feeling of oppression and alarm unconsciously felt by students involved in mathematical tasks. Math anxiety is known to impair cognitive faculties contributing to high performance in such tasks, such as working memory capacity [46]. Recent work indicates that math anxiety may be absent in home schooling situations compared to more stress-inducing face-to-face settings, even if students were enrolled in STEM-focused curricula [47]. Thus, homeschooling-related reductions in the performance gap may have been caused by greater reductions in math anxiety for low-performing compared to high-performing students. Finally, it is possible that students—especially low-performing students—may have been less distracted by other students, their teachers, or even potential stressful classroom settings classroom when learning at home, allowing them to focus better on their problem sets. If this were the case, then one would expect to observe benefits of homeschooling in other domains. However, previous studies suggest otherwise [8, 10, 48], showing that performance on national exams in the Netherlands decreased after the shutdown, as evidenced by a large dataset of approximately 350,000 K-12 students. These decreases were more severe for students from less educated families compared to educated families. Thus, the differential effects of school closures on students’ performance in national exams, on the one hand, and in online learning environments, on the other hand, demand further investigation, and may yield answers to important educational questions such as which learning environments are most suited for times in which students need to be taught from home.

In addition to fostering academic performance during school closures, online learning environments may hold promise for reducing performance differences between students, viz. the Matthew effect. Results from Analysis 2 suggest that low-performing students showed greater improvements in performance than high-performing students. One possible explanation for this effect is that online learning environments, like the one investigated in this study, allow teachers to adapt the assignment of problem sets according to students’ needs. Such individualization can be considered a graded form of tracking, that is, the separation of students into different learning groups based on their academic performance. Tracking has been identified as an effective measure to narrow educational gaps [49–52]. For instance, Duflo and colleagues (2011) [50] demonstrated in a field experiment, including over 300 first grade classes in Kenya, that the division of students into classes based on prior abilities can yield significant improvements in the academic performance of low-performing students. From this perspective, the individualization of problem sets in online-learning environments may complement a variety of other means that have been found to reduce differences in students’ performance and, ultimately, the Matthew effect [20, 21], including positive affirmations [53], the assignment of teachers to students based on teachers’ experience [54–57] or school vouchers [58].

While the present study provided first insights into the impact of school closures on the mathematical performance in an online-learning environment, future studies are needed to illuminate the factors that contributed to the reported improvements in performance. Future investigations may benefit from taking into account variables pertaining to teacher’s usage of online-learning environments, especially the use of incentives. As discussed above, we cannot rule out that teachers incentivized students differently during the pandemic as compared to the year before. Furthermore, as teachers did not use the software in an actual classroom setting during the shutdown of schools, it is unclear whether teachers adopted more/less formative homework, or more/less optional practice. In addition, students who participated in e-learning exercises may be more likely to afford a smartphone, tablet, or laptop. Students without access to e-learning platforms, e.g., due to a lower socio-economic status, may have received degraded teaching content, thus leading to worse overall as opposed to better performance. Therefore, the current results do not warrant conclusions about the influence of school closures on students without access to digital equipment, and/or access to internet.

In conclusion, the results from this study suggest that the shutdown of schools in 2020 had a positive impact on the performance of students in an online learning environment for mathematics, relative to the year before. Most importantly, we found that these improvements were greatest for students who performed below average in 2019. While future research is needed to contrast these findings with academic performance in traditional learning environments, the results of this study may help inform educators in identifying appropriate learning methods for home schooling situations. One can speculate that the supplemental use of online learning software, next to traditional learning materials, may turn out to be an effective teaching method, especially for narrowing performance gaps between students, during and beyond the ongoing pandemic.

## Supporting information

S1 File (DOCX)

S1 Data (XLSX)
