# Supplementary material for: Academic performance of K-12 students in an online-learning environment for mathematics increased during the shutdown of schools in wake of the COVID-19 pandemic
Source: PLoS One. 2021 Aug 3;16(8):e0255629. doi: 10.1371/journal.pone.0255629 (PMC8330947; doi:10.1371/journal.pone.0255629)
Supplement: S1 File — (DOCX) [file pone.0255629.s001.docx]

**S1 File**


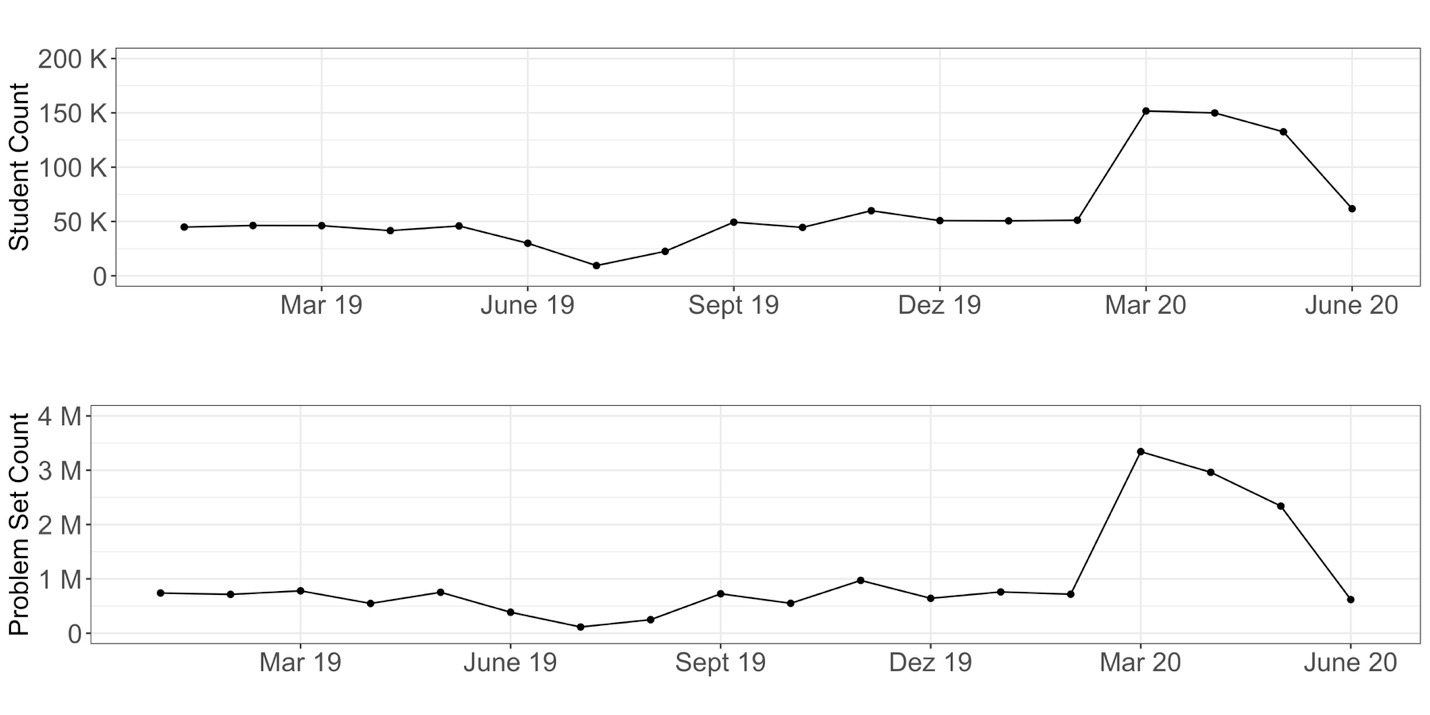


*S1 Fig*. **Count of students and computed problem sets in Germany** **as a function of time.** Upper panel: Each data point designates the number of students who computed problem sets with Bettermarks on a given month. Lower panel: Each data point indicates the number of problem sets computed with Bettermarks on a given month. Data included all students who used Bettermarks in Germany from January 1^st^, 2019 until June 15^th^, 2020.

**

*S2 Fig*. **Example exercises from different book topics.** Problems test a variety of mathematical competencies, including (1) plotting, (2) simplifying equations, (3) retrieval of mathematical laws and (4) solving equations.


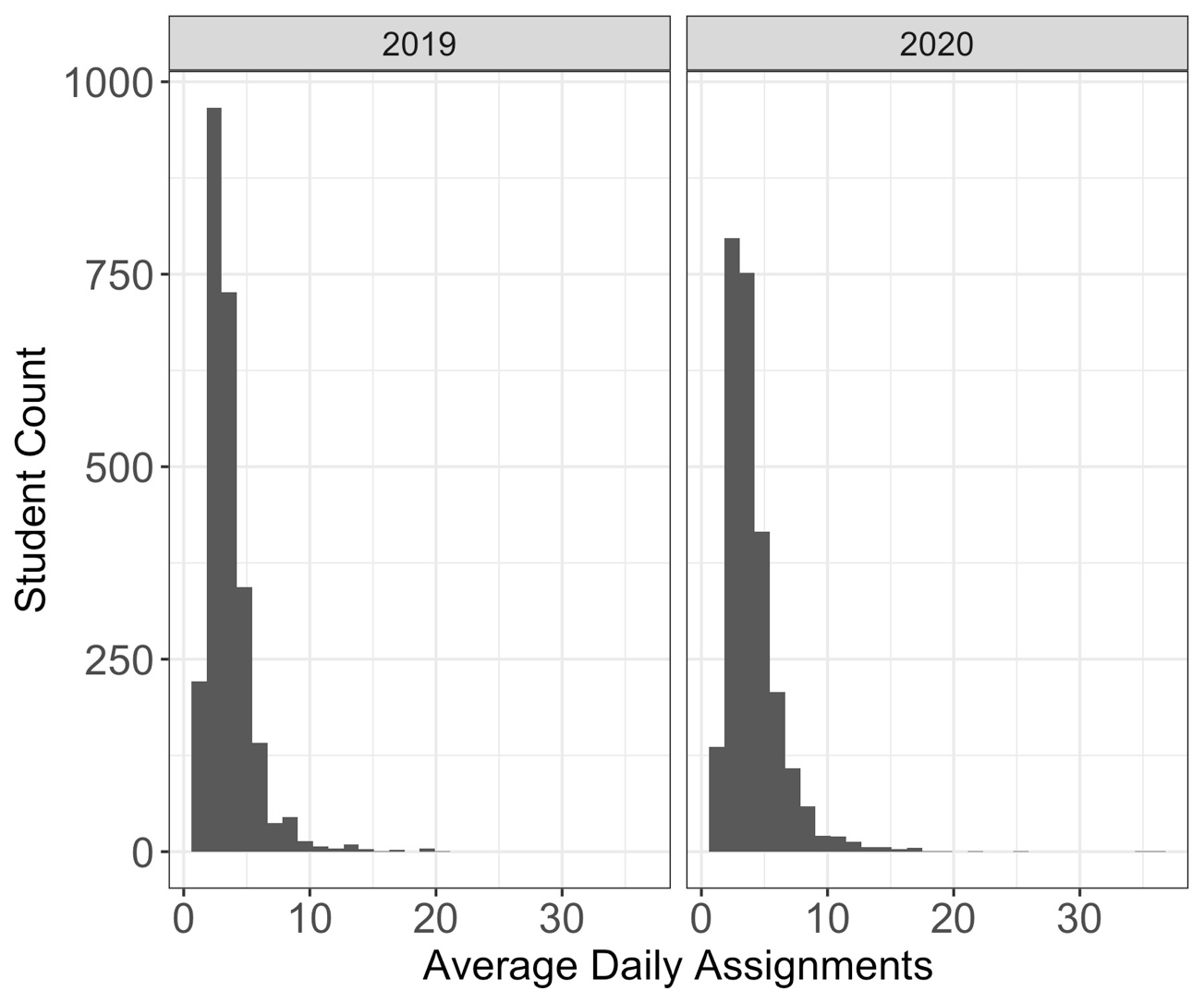


*S3 Fig*. **Histogram of average computed daily problem sets, separated by time window.** Each graph plots the total number of students computing a certain number of problem sets per day, averaged across days. Overall, students computed an average of 3.56 (*SD* = 1.90) problem sets in the 2019 time window and an average of 4.09 (*SD* = 2.37) problem sets in the 2020 time window.


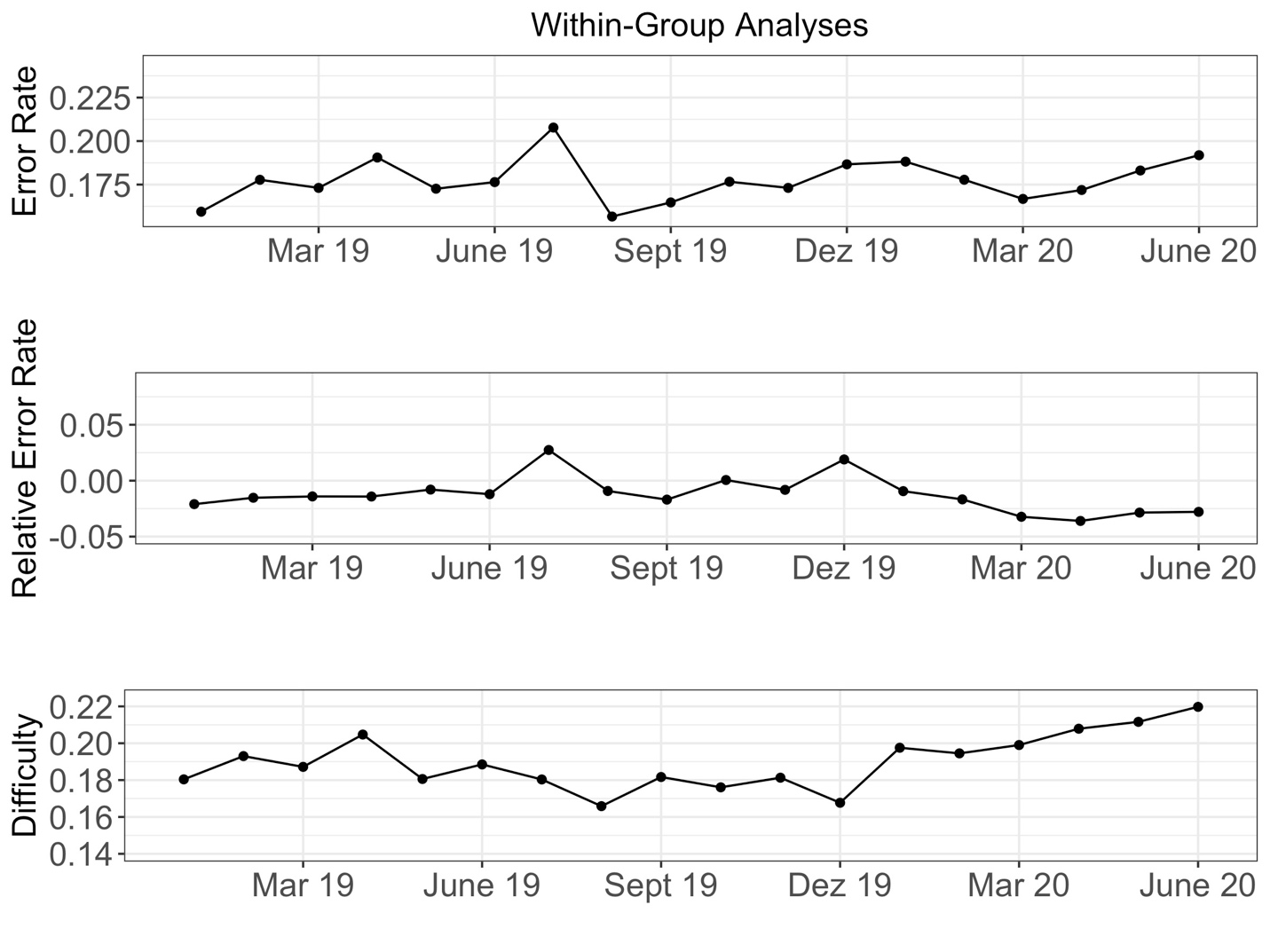


*S4 Fig*. **Average** **absolute** **error rates, relative error rates, and problem set difficulty as a function of time.** Both panels show data from students included in the within-group analysis (see text) who computed problem sets from January 1^st^, 2019 until June 15^th^, 2020. Each data point indicates the average statistic for a given variable on a given month. Variables were first averaged across problem sets for each student, and then averaged across students. The difficulty of a problem set corresponds to the average error rate of a reference group on that problem set (see text).
